# Copper homeostasis and the ubiquitin proteasome system

**DOI:** 10.1093/mtomcs/mfad010

**Published:** 2023-02-23

**Authors:** Bichao Zhang, Richard Burke

**Affiliations:** School of Life Science and Technology, Xinxiang Medical University, Xinxiang 453003, China; School of Biological Sciences, Monash University, Clayton 3800, Victoria, Australia

**Keywords:** copper homeostasis, copper transporters/chaperones, genetic interaction, neurodegenerative disease, ubiquitin proteasome system

## Abstract

Copper is involved in many physiological pathways and important biological processes as a cofactor of several copper-dependent enzymes. Given the requirement for copper and its potential toxicity, intracellular copper levels are tightly controlled. Disturbances of human copper homeostasis are characterized by disorders of copper overload (Wilson’s disease) or copper deficiency (Menkes disease). The maintenance of cellular copper levels involves numerous copper transporters and copper chaperones. Recently, accumulating evidence has revealed that components of the ubiquitin proteasome system (UPS) participate in the posttranslational regulation of these proteins, suggesting that they might play a role in maintaining copper homeostasis. Cellular copper levels could also affect the activity of the UPS, indicating that copper homeostasis and the UPS are interdependent. Copper homeostasis and the UPS are essential to the integrity of normal brain function and while separate links between neurodegenerative diseases and UPS inhibition/copper dyshomeostasis have been extensively reported, there is growing evidence that these two networks might contribute synergistically to the occurrence of neurodegenerative diseases. Here, we review the role of copper and the UPS in the development of Alzheimer’s disease, Parkinson’s disease, and amyotrophic lateral sclerosis, and discuss the genetic interactions between copper transporters/chaperones and components of the UPS.

## Graphical Abstract

**Graphical Abstract:**
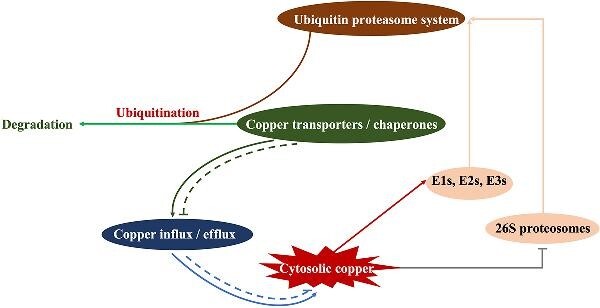
Summary of the overall interactions between the copper homeostasis machinery, cytosolic copper levels and the Ubiquitin Proteasomal System.

## Introduction

Copper is an essential trace element that is required as a catalytic cofactor and/or structural component of several important cellular enzymes involved in numerous biological processes including energy metabolism (e.g. cytochrome *c* oxidase), anti-oxidative defense (e.g. Zn, Cu-superoxide dismutase), and iron metabolism (e.g. ceruloplasmin).^1–3^ The relatively high redox potential of the Cu^2+/^Cu^+^ system is utilized for oxidation reactions, such as the generation of superoxide by superoxide dismutase (SOD) and catechols by tyrosinase.^4^ Since an excess of copper can also harm cells due to its potential to catalyze the generation of toxic reactive oxygen species (ROS),^5^ transport of copper and cellular copper content are tightly controlled.

One characteristic manifestation of copper dyshomeostasis is neurodegenerative disease, seen in classical Mendelian copper disorders such as Menkes disease, occipital horn syndrome (OHS), and Wilson’s disease (WD).^6–8^ The ubiquitin proteasome system (UPS) participates in the degradation of cytosolic, nuclear, and transmembrane proteins and plays an important role in neurodevelopment.^9,10^ Multiple UPS proteins have been demonstrated to interact with the copper homeostasis machinery. In this review, we survey the evidence that implicates the involvement of UPS in regulating cellular copper content and discuss the possibility of copper in turn influencing the UPS.

## Cellular copper homeostasis

**Fig. 1.**
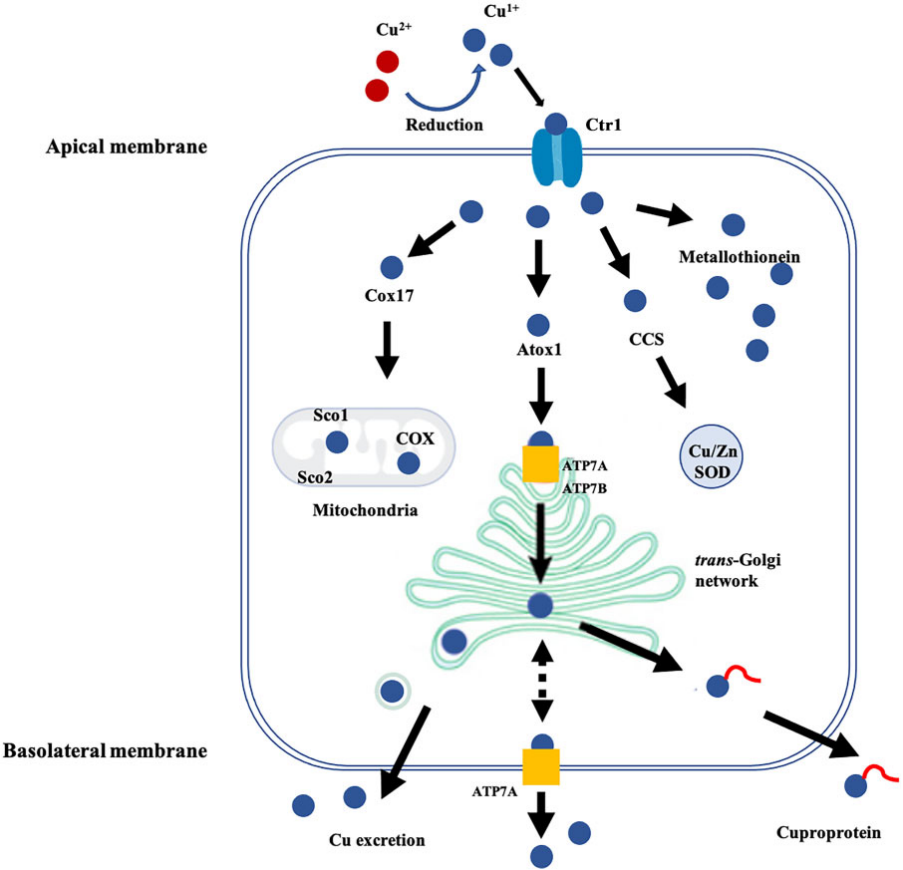
Model of cellular copper homeostasis, adapted from Davies *et al.*.^78^ In mammalian cells, Cu^2+^ ions are reduced to Cu^+^ before being imported into the cell by the copper import protein CTR1. Once Cu^+^ ions enter cells, they are distributed to different cellular locations by copper chaperones. Some are trafficked into the mitochondria for incorporation into COX by Cox17, Sco1, and Sco2. The copper transport protein Atox1 transports copper to ATP7A and ATP7B in the *trans*-Golgi network (TGN). CCS delivers copper to copper/zinc superoxide dismutase (Cu/Zn SOD/SOD1). Some free copper ions are sequestered by metallothioneins for maintaining copper homeostasis.

The copper homeostasis system is a complex network of proteins that bind and deliver copper to copper-dependent proteins and protect cells from the harmful effects of excess copper. Many of the components involved in cellular copper homeostasis have been identified, including transporters that mediate copper uptake and efflux, molecules that sequester and store copper, and chaperones that guide copper to copper-dependent enzymes, as shown in Fig. 1.

Members of the high-affinity copper uptake protein (CTR) family play a key role in copper uptake in eukaryotic cells.^11^ There are two CTR members in humans, CTR1 and CTR2. In vertebrates, CTR1 is expressed ubiquitously in all tissues.^12^ Structural studies reveal that CTR1 molecules are arranged in a symmetrical trimer, forming a pore for the passage of monovalent copper across the lipid bilayer.^13^ In polarized intestinal enterocytes, CTR1 was originally shown to be localized to the apical, luminal membrane.^14,15^ However, subsequent functional studies indicated that CTR1 mediates basolateral copper uptake (blood to intestinal cells) rather than apical transport (lumen to intestinal cells),^16^ a result supported by more recent studies of CTR1 intestinal knockout mice which also revealed that the original apical CTR1 signal may have come from non-absorptive epithelial cells.^17^ Thus, the main route of entry of dietary copper into the intestinal enterocytes remains unknown. CTR2 shares a high sequence similarity to CTR1.^12^ In multiple cells (HEK293T, Hela, U20S, and COS7), CTR2 has been detected in late endosomes and lysosomes,^18,19^ suggesting a role in copper recycling after degradation of copper enzymes. Even though one study has reported the presence of a small percentage of CTR2 (∼5%) at the plasma membrane (PM),^18^ whether CTR2 mediates copper uptake across PM as CTR1 does remains to be investigated.^20^

After being imported to cytosol, Cu^+^ ions are delivered to copper-dependent enzymes or sequestered, since free copper ions are harmful. The low concentration of free copper within the cell, less than one ion per cell, is maintained by the binding of copper to metallothioneins (MTs) and ligands of low molecular mass such as glutathione (GSH).^21^ The majority of cytosolic copper is bound to GSH,^22^ which can participate in cellular copper homeostasis by regulating the activities of the copper efflux proteins ATP7A and ATP7B via (de)glutathionylation.^23^ MTs are involved in the intracellular sequestration and storage of excess copper. In patients with WD, excess hepatic copper was found to be bound to MTs.^24^ An excess of copper induces the transcription of MTs,^25^ and therefore the rise in MT levels reflects an adaptation of cells to copper overload.

The intracellular trafficking of copper is mediated by a group of copper chaperones including antioxidant protein 1 (Atx1), copper chaperone for superoxide dismutase (CCS), and Cox17. Atox1, the human homologue of Atx1, has been demonstrated to bind Cu^+^ and transfer it to the transporters ATP7A and ATP7B, which deliver copper to cuproenzymes in the secretory pathway.^4^ CCS is primarily localized in the cytosol and its expression levels depend on cellular copper content.^26,27^ CCS transports copper imported by CTR1 to the cytosolic copper-dependent enzymes Cu, Zn-superoxide dismutase and BACE1.^26,28–30^ Cox17, a small, cysteine-rich, hydrophilic protein is responsible for delivering copper to mitochondrial cytochrome *c* oxidase via Cox11, Sco1, and Sco2.^31,32^

Cellular copper efflux in mammals relies on the function of two copper transporting P-type ATPases, ATP7A and ATP7B. ATP7A continuously recycles between the *trans*-Golgi network (TGN) and the PM, whereas ATP7B traffics between the TGN and a cytosolic vesicular compartment.^33,34^ ATP7A is ubiquitously expressed in mammals, and normally localized to the TGN where it is required for transporting copper to the Golgi lumen for incorporation into copper-dependent enzymes.^34,35^ When cells have excess copper, ATP7A-containing vesicles are transported to the PM for copper efflux.^36,37^ However, ATP7B is more highly expressed in the liver.^38^ Resemble to ATP7A, ATP7B normally resides in the TGN of hepatocytes for loading copper on cuproproteins.^39^ When intracellular copper is overloaded, ATP7B translocates to lysosomes for activating lysosomal exocytosis and then stimulating the release of copper into bile.^40^

## Copper dyshomeostasis

As a redox-active metal, copper participates in diverse metabolic processes in living organisms, but can also generate toxic ROS.^41^ In order to supply sufficient copper for the synthesis of cuproenzymes but also to prevent copper-induced oxidative stress, copper levels must be tightly regulated.^42^ Disturbance of copper homeostasis can cause both copper deficiency disorders [e.g. Menkes disease, OHS, distal hereditary peripheral neuropathy (dHMN)]^7,43^ and copper overload disorders (WD, copper-associated infantile cirrhosis).^8,44^

### Copper-deficiency disorders

Copper deficiency can occur through multiple mechanisms, such as low dietary copper intake or high zinc intake.^45,46^ The clinical symptoms of acquired copper deficiency in humans are numerous, including anemia and neuropathies.^47^ Severe copper deficiency is a hallmark of the X-linked recessive Menkes disease (MD), which is caused by genetic defects in the ATP7A copper transporting ATPase.^7^ MD is an incurable disorder that often leads to childhood mortality as a consequence of reduced copper efflux from the enterocytes into the bloodstream.^48–50^ The brain is especially sensitive to defective ATP7A protein and copper deficiency since the passage of copper across the blood–brain barrier (BBB) is mediated by ATP7A, and several cuproenzymes (e.g. Cu, Zn-superoxide dismutase) play critical roles in neuronal development.^7,51–53^ Paradoxically, although less copper is transported to the blood and major tissues including the brain resulting in neurological and connective tissue abnormalities, copper accumulates in other tissues of MD patients such as the duodenum, kidney, spleen, pancreas, and skeletal muscles.^54,55^

Specific missense mutations in ATP7A also cause other copper deficiency disorders such as occipital OHS^56^ and dHMN.^6,43^ OHS, caused by splice site mutations in *ATP7A* gene, is recognized the mildest form of MD, leading to a connective tissue disorder resulting from lowered activity of lysyl oxidase.^56–58^ The splice site mutations lead to the generation of only a small amount of normal ATP7A protein, thereby, decreasing copper efflux.^58^ Mercer *et al*. postulated that normal ATP7A would be translocated to the PM even in quite low copper concentrations, which generates ATP7A deficiency in the TGN and subsequent lack of copper supply to lysyl oxidase (reduced activity).^56^ Distal hereditary peripheral neuropathy is also known as spinal muscular atrophy, characterized by distal muscle weakness and wasting caused by degeneration of spinal motor neurons.^59^ Two unique ATP7A missense mutations (p.P1386S and p.T994I) were identified in males with dHMN, especially p.P1386S, which resulted in a defect in ATP7A trafficking although ATP7A mRNA and protein levels remained normal.^43^

### Copper overload disorders

Copper in excess is highly toxic. WD is a serious but mostly treatable copper toxicity disorder caused by recessive mutations in ATP7B.^8^ ATP7B, structurally similar to ATP7A, is mainly restricted to the liver where it is required for exporting copper to the bile.^60^ In WD, copper accumulates in the liver due to the failure of ATP7B-mediated copper efflux, compromising hepatocyte function and leading to the unregulated release of copper, which accumulates in the central nervous system resulting neurological abnormalities.^8^ This disease can be treated by reducing systemic copper levels either by using copper chelators or blocking intestinal copper uptake with excess zinc supplementation.^44^

## Copper homeostasis and the UPS

**Fig. 2.**
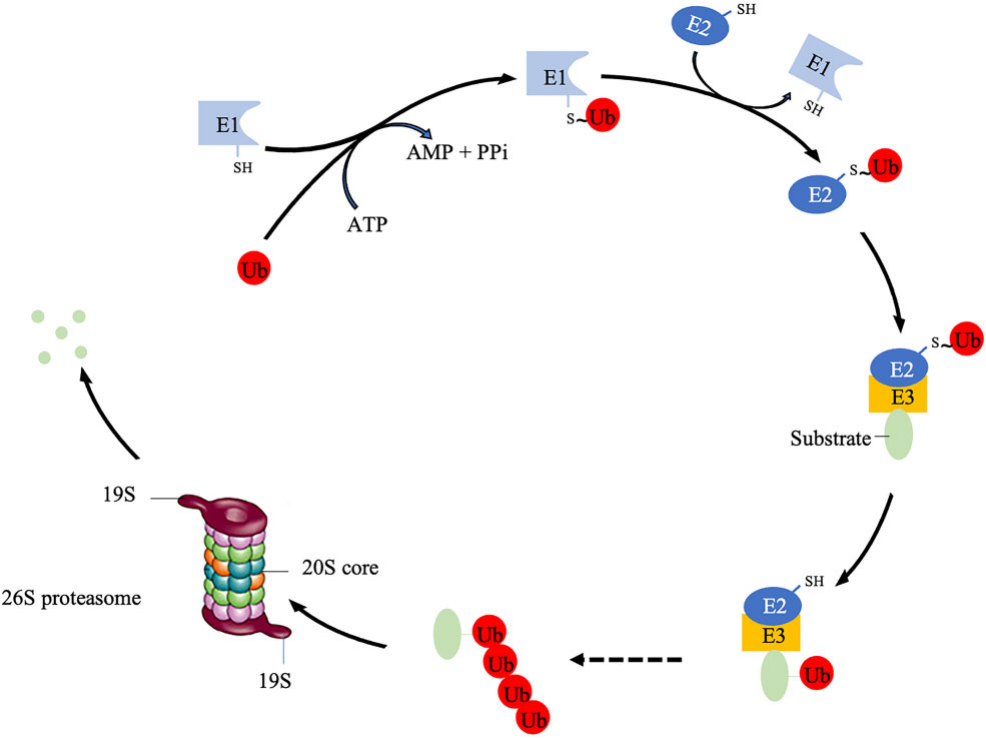
Ubiquitination and proteasomal degradation, adapted from Maupin-Furlow.^62^ Ubiquitination is a common signal for degradation of tagged proteins by the eukaryotic 26S proteasome and involves a cascade of E1 ubiquitin-activating, E2 ubiquitin-conjugating and E3 ubiquitin ligase enzymes.

The UPS is a selective proteolytic mechanism for degrading ubiquitin-conjugated substrates by the proteasome.^61^ Ubiquitination also serves as a signal to control the activation of multiple intracellular signaling pathways. Three key enzyme types participate in ubiquitination: E1 ubiquitin activating enzymes, E2 ubiquitin conjugating enzymes, and E3 ubiquitin ligases.^61^ The process of ubiquitin-mediated substrate delivery to 26S proteasomes is summarized in Fig. 2. Ubiquitin is initially activated by an E1 in the presence of ATP and then transferred to an E2 through a thioester bond. An E3 subsequently catalyzes the transfer of ubiquitin from E2 to Lys residues within specific substrates. Once the substrate is tagged with at least four ubiquitin molecules, it will be recognized by the 26S proteasome for degradation.^62,63^

In humans, more than 40 E2s and 500 E3s have been identified to date.^64,65^ In contrast, the human genome encodes just nine E1 enzymes.^66^ E3s play a critical role in target selection and specificity.^63^ They are categorized into four major classes based on specific structural motifs: HECT-type; RING-finger-type; U-box-type; and PHD-finger-type. RING-finger-type E3s constitute the largest family and cullin-based E3s are one of its largest subfamilies.^65^ There are seven cullin-based E3s including the SKP1–CUL1–F-box-protein (SCF) complex, which consists of three invariable components, RBX1 (a RING-finger protein), CUL1 (a scaffold protein), and SKP1 (an adaptor protein) as well as one variable component—the F-box protein.^67^ The F-box protein binds to an F-box motif within SKP1 and is responsible for substrate recognition.^65^

The UPS is involved in numerous biological process including: (i) regulation of the cell cycle;^68^ (ii) cancer and cell survival;^69^ (iii) inflammatory responses;^70^ (iv) immune response;^71^ (v) degradation of misfolded proteins;^72^ and (vi) endogenous reticular associated degradation.^73^ Therefore, it is unsurprising that UPS dysregulation is heavily implicated in disease progression^74,75^ and targeted manipulation of the UPS is seen as a promising therapeutic strategy.

### The UPS, copper homeostasis, and neurodegenerative diseases

The UPS is considered a master regulator of neural development and maintenance of brain structure and function^10^ and UPS dysfunction has been linked to aging and the development of several neurodegenerative diseases, as has copper dysregulation. Here, we review the evidence of UPS contributions to Alzheimer’s disease (AD), Parkinson’s disease (PD), and amyotrophic lateral sclerosis (ALS), three disorders frequently associated with altered copper homeostasis.^76–78^ We then explore the extensive links between these diseases and UPS/copper.

#### The role of the UPS and copper in AD

**Fig. 3.**
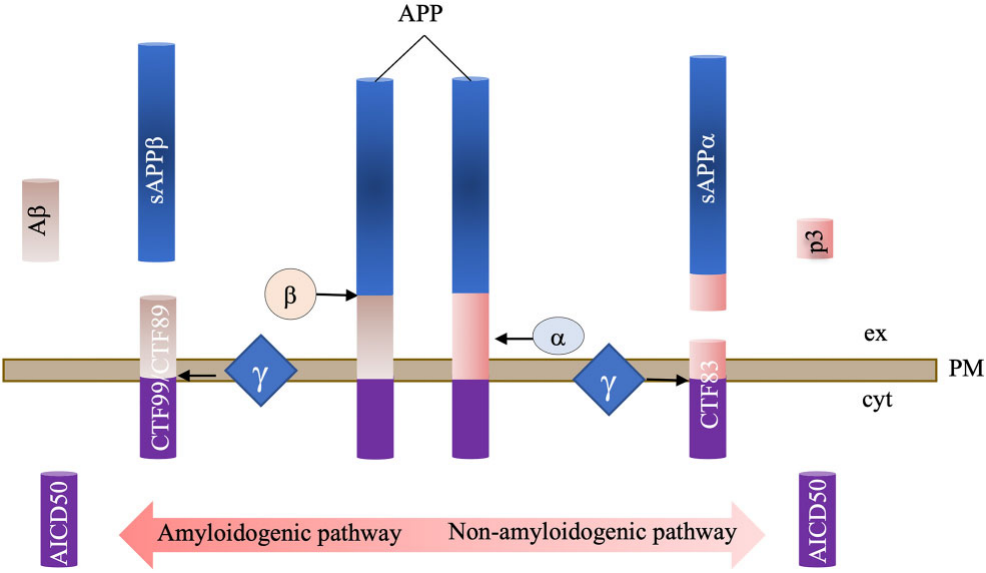
APP processing and cleavage products. The non-amyloidogenic pathway involves the initial cleavage of APP via α-secretase, followed by γ-secretase-mediated cleavage, generating a long-secreted form of APP (sAPPα) and C-terminal fragments (CTF 83, p3 and AICD50).^229–233^ In the amyloidogenic pathway, APP is sequentially cleaved by β-secretase (BACE1) and γ-secretase, which results in the generation of a long-secreted form of APP (sAPPβ), C-terminal fragments (CTF 99 and CTF 89) and Aβs.^234^ Aβ fragments oligomerize and fibrillize leading to AD pathology (left and upper panel). ex, extracellular; PM, plasma membrane; cyt, cytosol.

AD is a progressive brain disorder where dementia symptoms gradually worsen over time. Amyloid precursor protein (APP), a transmembrane glycoprotein, plays important roles in neuronal plasticity and brain homeostasis.^79,80^ APP can be proteolytically processed via both amyloidogenic and non-amyloidogenic pathways (Fig. 3). Amyloid-β (Aβ) peptide, generated via the amyloidogenic pathway, is thought to be the causative factor of AD.^81^ Excessive Aβ accumulation and deposition may trigger a complex downstream cascade that results in the symptoms of AD.^82,83^ Aβ peptides are deposited in amyloid plaques, the pathological hallmarks found in the central nervous system of AD patients.^84–86^ Aβ40 represents the most common amyloid species overall while Aβ42 is the most abundant species in amyloid plaques.^87^ Aβ42 peptides can aggregate to oligomers which mediate neurotoxicity and participate in the formation of amyloid plaques and neurofibrillary tangles (NFTs).^88,89^ In particular, Aβ42 production can be augmented by mutations in three different genes that cause familial forms of AD, APP, presenilin-1 (PS1), and presenilin-2 (PS2).^90^ There is increasing evidence that disruption of UPS activity is implicated in the accumulation of Aβ42 peptide in AD.^91^ Therefore, we reviewed the role of UPS in the generation of Aβ42 oligomers.

The UPS has been found to play a critical role in maintaining Aβ42 equilibrium via posttranslational regulation. In primary cultures of cortical neurons and astrocytes, inhibition of 26S proteasome activity by lactacystin (a non-peptidic proteasome inhibitor) resulted in a remarkable decrease in Aβ42 degradation.^89^ In addition, in Aβ42 overexpressing cells treated with MG132 (a cell-permeable proteasome inhibitor), monomeric Aβ42, low molecular weight (MW) Aβ42 oligomers, putative oligomeric 6 kDa and trimeric 12 kDa Aβ42 levels were dramatically elevated while the levels of high-MW Aβ42 oligomer were not significantly altered. These data suggest that the UPS preferentially removes monomeric Aβ42 and low-MW Aβ42 aggregates,^92^ explaining why inhibition of UPS activity could lead to the accumulation of Aβ42. In the hippocampus and cortex of AD patients, the E3 ubiquitin ligase Parkin was found to colocalize with intraneuronal Aβ42^93^ and in a triple transgenic AD mouse, Parkin expression was shown to decrease Aβ levels and extracellular plaque deposition,^94^ suggesting it may mediate at least some of the UPS's Aβ42 clearing activity.

The activity of Parkin in the clearance of ubiquitinated Aβ in AD models involves beclin-dependent autophagy.^93,94^ A further link with copper homeostasis was thus revealed by the recent finding that intracellular copper can stimulate autophagy in lung adenocarcinoma cells by enhancing activity of the autophagic kinases ULK1 and ULK2^95^ through direct binding of copper to these proteins. These results add further support to the growing body of literature supporting the use of copper chelation in cancer therapy and it will be fascinating to see if this ULK1/2-mediated relationship between copper and autophagy can also be observed in neuronal cells.

The UPS also has the potential to regulate Aβ production via two key secretases in the amyloidogenic pathway, β-secretase (BACE1) and γ-secretase.^81^ BACE1 in particular plays a central role in β-amyloidogenesis (see Fig. 3). Fbx2, a neuron-specific F-box protein, forms the SCF^Fbx2^-E3 ubiquitin ligase complex by binding to the Skp1 domain of SCF (Skp1–Cullin1–F-box protein).^81^ The SCF^Fbx2^-E3 ligase binds and ubiquitinates BACE1 via the Trp 280 residue of its F-box-associated domain. In the brain of AD-model mice, BACE1 protein levels and activity were reduced by overexpressing Fbx2, resulting in a decrease in Aβ levels.^96^

DYRK1A, a member of the dual specificity tyrosine phosphorylation-regulated kinase family, has been reported to be involved in the progression of Down syndrome (DS).^97^ DS is an useful model for AD research since the majority of DS individuals show features of AD because APP is located on chromosome 21.^98^ DYRK1A was demonstrated to phosphorylate APP on Thr-668, which then facilitated the BACE1-mediated cleavage of APP, leading to an increase of Aβ.^99^ In HEK293 cells, Co-IP assay results revealed that SCF^βTrCP^-E3 ligase and DYRK1A could be precipitated simultaneously by βTrCP antibody while knockdown of βTrCP expression with siRNA significantly increased DRYK1A protein levels, indicating that SCF^βTrCP^-mediated ubiquitination and promoted degradation of DYRK1A,^100^ adding to the role of the UPS in influencing APP processing.

γ-secretase, the second secretase involved in β-amyloidogenesis, is made up of four subunits, nicastrin (NCT), PS (two isoforms PS1 and PS2), PS enhancer-2 (PEN-2) and anterior pharynx defective-1 (APH-1).^101,102^ These four components are necessary and sufficient for γ-secretase activity in *Caenorhabditis elegans, Drosophila*, yeast, and mammalian cells.^66^ High levels of ubiquilin (ubiquitin-like protein) were found to reduce γ-secretase activity by decreasing the formation of PS fragments and/or the levels of PEN-2 and NCT.^81^ Furthermore, specific transcript variants of ubiquilin-1 which are genetically and functionally associated with AD, regulate proteasomal and aggresomal targeting of PS1.^101,103^ In addition, inhibition of proteasomal degradation of APH-1 by lactacystin in N2a cells facilitated γ-secretase-mediated cleavage of APP to generate Aβ.^66^ These studies demonstrated that the UPS regulates γ-secretase activity via post-translational regulation of its four components, thereby contributing indirectly to the regulation of Aβ42 generation.

Hyperphosphorylation of the microtubule binding protein tau is also considered as a hallmark of AD. Tau phosphorylation abnormalities have been linked to misfolding and deposition of the protein in NFTs.^104,105^ Immunoblot results revealed that overexpression of the E3 ubiquitin ligase carboxyl-terminus-of-HSP70-interacting protein (CHIP) in COS7 cells induces the ubiquitination of phosphorylated tau in collaboration with E2 ubiquitin conjugating enzyme UbcH5B,^106^ suggesting the importance of UPS in maintaining tau equilibrium.

Given that the inhibition of the UPS activity is associated with the accumulation of Aβ42 oligomers and misfolded tau, enhancing UPS function has been put forward as a promising therapeutic strategy for AD. One emerging approach is the targeted degradation of specific molecules by proteolysis targeting chimeric molecules (PROTACs).^107^ Theoretically, PROTACs could be developed for the degradation of proteases involved in Aβ42 production or misfolded tau.^91^ However, the impact of Aβ and tau on the efficacy of PROTACs in AD needs to be overcome for this approach to be effective. The association of transition metals such as zinc, copper, and iron with the pathophysiology and neuropathology of AD has been thoroughly reviewed by Lei *et al*.^108^ The AD brain is characterized by abnormal neuronal copper distribution, with accumulation of copper in amyloid plaques and deficiency in the cells.^108–110^ Lovell *et al*. reported a significant elevation of copper levels in the core of senile plaques (also known as amyloid plaque) in patients with AD using micro particle-induced X-ray emission,^109^ further evidenced by Miller *et al*.^111^ In an APP/PS1 mouse model of AD, X-ray fluorescence microscopy and immunohistochemical staining showed an increase of copper levels in Aβ plaques.^112^ However, the total copper in AD brain was substantively decreased, compared with the healthy control.^113,114^ Therefore, neither copper chelation nor copper supplementation could achieve ideal benefits for AD.

Copper was found to inhibit the ability of γ-secretase to cleave APP-C99 and form Aβ via directly binding to its subunit NCT and PS [115]. Human embryonic kidney cells exposed to high copper conditions inhibited Aβ production, whereas copper deficiency in human neuroblastoma cells dramatically elevated Aβ production.^110^ In the TgCRND8 APP transgenic mice, elevation of intracellular copper levels by ATP7B mutation reduced plasma Aβ.^116^ It is implied that Aβ formation is subject to the alteration of intracellular copper level. Aβ possesses a copper binding site which only emerges once the carboxyl terminus is cleaved from APP.^108,117^ Atwood *et al*. demonstrated that Aβ42 has a high affinity Cu^2+^ binding site which mediates peptide precipitation, and the presence of Cu^2+^ resulted in the self-aggregation of Aβ42.^118^ In a *Drosophila* Aβ transgenic model, lowering copper levels by suppressing Ctr1B (the *Drosophila* homologue of CTR1) or overexpressing ATP7 (the *Drosophila* homologue of ATP7A and ATP7B) resulted in the decrease of Aβ oligomers.^119^ These data support the proposition that intracellular copper deficiency increases Aβ production while the accumulation of extracellular copper promotes Aβ deposition.^108^

**Fig. 4.**
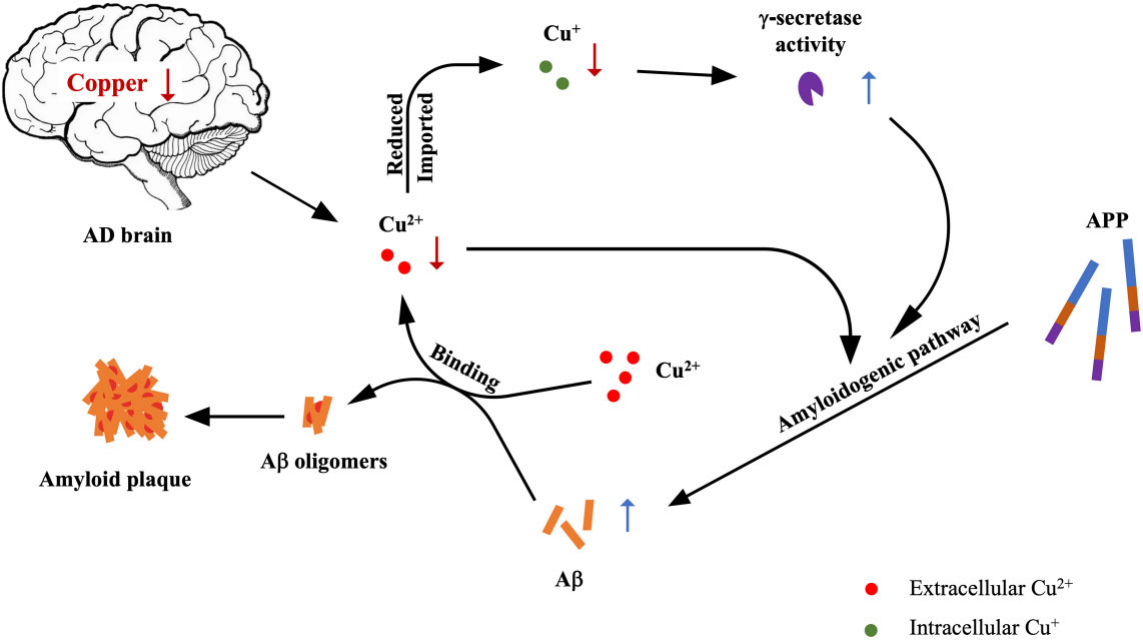
The association of copper with Aβ production and aggregation in AD. In AD brain tissue, intracellular copper deficiency promotes the generation of Aβ via the amyloidogenic pathway, whereas extracellular Cu^2+^ co-aggregates with Aβ to form amyloid plaques.

APP itself possesses an N-terminal copper binding region capable of reducing Cu^2+^ to Cu^+^.^87^ Cu^2+^ ions binding to APP could cause a conformational change, which then facilitates α-secretase-mediated cleavage but attenuates BACE1-mediated cleavage, thus promoting the non-amyloidogenic APP processing pathway.^120^ This discovery provides another mechanism by which copper inhibits Aβ production. Based on these findings, we proposed a hypothesis about the association of copper with Aβ production and aggregation in AD (Fig. 4). Decreased brain copper levels have been detected in AD postmortem brain samples,^114^ indicating that free extracellular Cu^2+^ is insufficient, which triggers the BACE1-mediated cleavage of APP, consequently increasing Aβ production. Meanwhile, less Cu^2+^ is reduced to Cu^+^ and then pumped into cells by CTR1, which removes the inhibition effect of Cu^+^ on γ-secretase activity and promotes the generation of Aβ. Thus, these two pathways together lead to the increase of Aβ secretion into the extracellular matrix. Excess Aβ binds extracellular Cu^2+^, which further depletes free Cu^2+^, and copper/Aβ co-aggregate to form amyloid plaques, explaining why copper accumulates in senile plaques despite the decrease in total copper in AD brain [108]. Hence, an ideal agent for alleviating AD would dissociate copper from amyloid plaques while mobilizing it into the cells.

The extensive links between copper dyshomeostasis and multiple aspects of AD have led to research on the potential of copper-modulating drugs to treat this disease. *In vitro* and mouse model studies revealed that metal protein attenuating compounds (MPACs) might promote the solubilization and clearance of Aβ42.^121^ Clioquinol (5-chloro-7-iodo-8-hydroxyquinolinol, also known as PBT1), a class of MPACs, which has affinity for copper/zinc ions and can cross the BBB, was reported to inhibit the formation of amyloid plaques in a mouse AD model and to dissolve Aβ deposits in postmortem human tissues.^122–125^ Therefore, it has been applied to the treatment of AD. In one trial, the rate of cognitive decline in AD patients treated with PBT1 was significantly retarded, compared with placebo group.^126^ However, this effect was just seen in the more severely affected patients at weeks 4 and 24, but not maintained at week 36. No current evidence leads to the conclusion that PBT1 has any significant effect on cognition in human patients with AD. Furthermore, PBT1 has been abandoned due to toxic impurities.^127^ PBT2, a derivative of clioquinol, has been clinically tested in human trials where it was shown to outperform PBT1 and improved cognitive performance.^128^ The following trials showed satisfactory safety and tolerability of PBT2 in AD patients.^127^ However, larger and longer future trials will be required to establish any potential therapeutic benefits.

Cu^2+^ complexes of bis-thiosemicarbazones, especially Cu(gtsm), have been demonstrated to increase intracellular copper levels and decrease secreted Aβ levels in APP-CHO cells.^129^ APP/PS1 transgenic AD mice treated with Cu(gtsm) showed lower abundance of Aβ trimers and phosphorylated tau.^130^ The underlying mechanism might be that Cu^2+^ ions binding to APP promote the non-amyloidogenic APP processing pathway and therefore decrease the production of Aβ.

#### The role of the UPS and copper in PD

PD is a long-term neurodegenerative disorder, characterized by a selective loss of dopaminergic neurons in the *substantia nigra* region of the brain and the presence of Lewy bodies (LBs).^131^ α-synuclein (α-Syn) is a natively unfolded presynaptic protein considered as a major pathogenic factor in PD due to its accumulation in LBs or Lewy neurites.^132,133^ Unbound and cytosolic α-Syn is a substrate for the proteasome while membrane-bound α-Syn is protected against proteasomal degradation.^134^ High levels of undegraded or poorly degraded α-Syn protein tend to self-aggregate, induce aggregation of other proteins, interfere with intracellular functions and induce cytotoxicity.^135^ In *Drosophila*, exogenous expression of *α-Syn* produces adult-onset loss of dopaminergic neurons, the formation of filamentous intraneuronal inclusions and locomotor dysfunction.^136^ In mice, ablation of α-Syn alleviates the symptoms of Parkinson-like syndrome induced by the dopaminergic neurotoxin 1-methyl-4-phenyl-1,2,3,6-tetrahydropyridine (MPTP), such as neuronal loss and the formation of ubiquitin-positive inclusions.^137^

The E3 ubiquitin ligase Parkin has also been linked to PD. Mutations in the *parkin* gene are a major cause of early-onset autosomal recessive familial PD and isolated juvenile-onset PD.^138^ Parkin may normally protect cells from premature death by degrading misfolded or damaged proteins.^139^ Parkin has been shown to become more insoluble with age and PD caused by Parkin mutations appears to universally result from alterations in Parkin solubility and intracellular localization.^139,140^ In PD brains, Parkin has been found to colocalize with α-Syn in LBs, even though α-Syn is not a substrate of Parkin.^140,141^ α-Syn and Parkin have been shown to associate and colocalize to cytosol and neuritic processes in PD.^142^ α-Syn aggregates interfere with Parkin solubility and distribution; co-expression of α-Syn and Parkin led to a decrease in Parkin solubility while α-Syn knockdown increased Parkin solubility.^143^

Heat shock proteins (HSPs) such as HSP70 and HSP90 also participate in the management of excess or deleterious proteins^144^ and CHIP, an E3 ubiquitin ligase, can mediate the degradation of misfolded proteins associated with PD.^145,146^ CHIP can rescue the cytotoxicity caused by α-Syn oligomers and reduce the formation of higher MW oligomeric α-Syn species.^146^ Bcl-2-associated athanogene 5 inhibits CHIP-mediated ubiquitination of α-Syn, and regulates the ability of CHIP to decrease the levels of α-Syn oligomeric species.^147^ Since the UPS plays a critical role in removing α-Syn aggregates, understanding the mechanism by which it achieves this could provide a promising therapeutic avenue for PD.

The cytotoxic aspects of copper are usually seen when it is present as a free ion or linked to low MW ligands. In PD, free copper is associated with increased oxidative stress, α-Syn oligomerization and LB formation.^148^ α-Syn has high affinity for copper through two binding sites, M1-D2 and H50.^149–151^ The binding of copper to α-Syn is an important event for the development of PD.^148^ Copper has been demonstrated to accelerate the formation of toxic oligomeric forms of α-Syn.^152–155^  *In vitro* studies revealed that copper and dopamine cooperatively bind to α-Syn at different sites and enhance the propensity of α-Syn to oligomerize.^156^ In a human blastoma cell line, copper depletion results in the redistribution of α-Syn toward the PM and reduces aggregate formation.^152^ Quantum and molecular mechanics simulations revealed that copper binding to α-Syn could make α-Syn more susceptible to misfolding which might then result in the formation of LBs.^157^ These results demonstrated that copper dyshomeostasis can contribute to the development of PD.

Some copper binding proteins are also found to be involved in the progress of PD. Ceruloplasmin, a multicopper-containing glycoprotein, has two prominent functions: plasma copper binding (95% of circulating copper is bound to ceruloplasmin); and regulating iron homeostasis by means of its ferroxidase activity.^148^ In the brain, ceruloplasmin is synthesized by astrocytes and is linked to iron efflux from the brain since ceruloplasmin promotes the oxidation of neuronal Fe^2+^ to Fe^3+^, which can then be exported.^148,158–160^ The ferroxidase activity of ceruloplasmin is diminished by severe copper deficiency.^148^ In the plasma and cerebrospinal fluid of patients with PD, copper-dependent ferroxidase activity has also been reported to be diminished. Furthermore, the postmortem basal ganglia and *substantia nigra* of PD patients both showed increased iron and decreased copper.^161–163^ Ceruloplasmin-deficient mice displayed neuronal cell death in the *substantia nigra*, which could be partially rescued by an iron chelator.^163^ Additionally, the increase of iron in the *substantia nigra* and the dopaminergic cell death induced by MPTP were partially prevented by the peripheral administration of ceruloplasmin.^163^ These suggest that the ferroxidase activity of ceruloplasmin is diminished by copper deficiency, which then causes the accumulation of iron and promotes the development of PD.

An additional link between PD and copper homeostasis is found through SOD1. SODs are major antioxidant enzymes responsible for eliminating superoxide anion radicals,^164^ and they have consistently been found to be neuroprotective.^148^ Three distinct isoforms of SOD have been identified in mammals: Cu, Zn-SOD (SOD1), Mn-SOD (SOD2), and extracellular-SOD (SOD3).^2^ SOD1 requires copper and zinc as cofactors, and has an important function as a copper buffer within cells.^165,166^ In patients with PD, SOD1 was diminished in red blood cells which resulted in a higher concentration of hydroxyl radical in the plasma.^167^ In addition, SOD activity is reduced significantly as the disease progresses, indicating the age-dependent deterioration of the antioxidant ability of SOD1 in PD.^168^

Radiolabeled Cu(atsm) [Cu-diacetyl-bis(N^4^-methylthiosemicarbazone)], is a promising marker for intracellular states for disorders with mitochondrial dysfunction, including PD.^169^ Recently, unlabeled Cu(atsm) has been shown to be effective in reversing parkinsonian defects in animal models. In multiple PD mouse models (e.g. MPTP-lesioned mice, hA53T α-synuclein tg mice), Cu(atsm) treatment improved motor and cognition function, rescued nigral cell loss and improved dopamine metabolism.^170^ It was also found to inhibit the formation of α-Syn oligomers because of its ability to suppress ONOO^−^-mediated nitration which induces α-Syn aggregation.^170,171^ Furthermore, Cheng *et al*. identified that the expression of 40 genes involved in dopamine synthesis and synaptic plasticity could be restored by Cu(atsm) treatment in MPTP induced PD mice.^172^ These studies suggest Cu(atsm) may serve as a promising neuroprotective drug to ameliorate the defects of PD.

#### The role of the UPS and copper in ALS

ALS is characterized by the progressive degeneration of motor neurons in the brain and spinal cord associated with the accumulation of misfolded proteins and insoluble inclusions.^173,174^ This protein misfolding disorder can be divided into sporadic and familial ALS.^173^ The most common genetic form of familial ALS is due to mutations in the SOD1 because they tend to be misfolded and form protease-resistant aggregates.^175,176^

Misfolded SOD1 is initially targeted for degradation by components of the UPS such as chaperones and ubiquitin ligases.^61^ However, mutant SOD1 tends to aggregate and escape the delivery process to proteasomes. In SOD1G93A transgenic mice (a familial ALS mouse model), mutant SOD1 accumulates in the cytoplasm of motor neurons, and then proceeds to form numerous inclusions in the axons and astrocytes.^176^ Large inclusions are clinical hallmarks of ALS symptoms. Furthermore, intracellular inclusions containing ubiquitin and ubiquitin ligases were detected in familial ALS mutant mice, suggesting that mutant SOD1 is resistant to the UPS.^177^ Additionally, overexpressing mutant SOD1G93A in mice stimulates the formation of SOD1 aggregates and inhibits proteasome activity; the formation of SOD1 aggregates is reversible with the restoration of proteasome function.^178^ Moreover, it has been shown that reduced proteasomal activity can promote the accumulation of ALS protein aggregates^177^. Inhibition of the proteasome by lactacystin causes the formation of SOD1 aggregates in SOD1 mutant expressing cells,^179^ suggesting that UPS plays a critical role in removing misfolded SOD1 in ALS.

Copper is required for SOD activity^180^ and SOD1 mutant mice showed elevated concentrations of copper in the spinal cord during disease progression, suggesting that copper dyshomeostasis might facilitate the development of ALS.^181^ Overexpressing the copper chaperone CCS, which delivers copper to SOD1, led to an accelerated pathology and disease progression in the SOD1 G93A mice.^182^ Moreover, copper deficiency has been demonstrated to accelerate aberrant hydrophobicity of both wild-type and mutated SOD1 due to partial protein unfolding, which could be reverted by the addition of Cu^2+^.^183^ Several studies have shown that mutant SOD1 aggregates in cultured cells or SOD1 transgenic mice have a low copper content.^184–186^ These results revealed that SOD1 mutant proteins accumulate in a copper-deficient state, even though overall copper levels may be elevated.

Cu(atsm) has been utilized for imaging in ALS but also has potential as a therapeutic.^187^ Cu(astm) administration to ALS mouse models (SOD1G93A and SOD1G37R mice), resulted in delayed onset of paralysis, preserved motor neurons and extended survival.^188,189^. In the spinal cord, SOD1 activity was enhanced in Cu(atsm) treated mice.^188^ These results imply that Cu(atsm) might prevent the accumulation of mutated SOD1 by increasing the formation of physiologically mature SOD1 via a copper delivery mechanism [Cu(atsm) delivers copper to stabilize the mutant SOD1 in a physiological holo form].^187^

### Copper homeostasis and UPS

The disruption of both copper levels and the UPS is detected in neurodegenerative diseases such as AD, PD and ALS, while a growing body of results has demonstrated that they both are associated with the generation or aggregation of pathogenic Aβ42, α-Syn, and SOD1, respectively. These commonalities suggest that the copper homeostasis machinery and the UPS may interact during the development of neurodegenerative disease. Therefore, we next review evidence for the interaction between the UPS and the copper homeostasis machinery.

#### Copper transporters/chaperones and the UPS

To date, several studies have revealed that components of the UPS participate in copper homeostasis directly or indirectly via copper transporters or copper chaperones. In yeast, the E3 ubiquitin ligase Rsp5 participates in the proteasomal degradation of copper import protein CTR1, thus decreasing cellular copper levels due to the reduction of copper uptake.^190^ NEDD4-L, the mammalian homologue of Rsp5, was also reported to regulate the degradation of CTR1 in breast cancer.^191^ Our research group has identified two E3 ubiquitin ligases which indirectly regulate Ctr1A, the *Drosophila* orthologue of CTR1: supernumerary limbs (Slmb) and von Hippel–Lindau (VHL) protein.^192,193^ The *Slmb* gene encodes a F-box protein orthologous to the human b-TrCP/BTRC protein, which binds with Skp1 and Cul1 to form a Skp1–Cul1–F-box (SCF) E3 ubiquitin complex. Knockdown of *Slmb* produced copper deficient defects due to the decrease of Ctr1A which could be rescued by co-knockdown of the transcription factor Cap-n-Collar (cnc, Nrf2 in mammals), a known substrate of Slmb/BTRC. We proposed that Slmb indirectly mediates the expression of Ctr1A via cnc, which then regulates cellular copper levels.^192^

VHL encodes a tumor suppressor, which possesses E3 ubiquitin activity in complex with the scaffold proteins EloB/C, Cul2 and Rox1a/Rbx1.^194^ In clear cell renal cell carcinoma, VHL activity is suppressed, resulting in the accumulation of the transcription factor Hif-1α in response to hypoxia.^195^ Hypoxia has been found to stimulate copper uptake via increasing CTR1 expression through the hypoxia-induced factor pathway.^196–198^ In *Drosophila*, we found that Vhl functions differently in different tissues, influencing melanin formation by altering copper levels via Ctr1A. The transcription factor involved in Vhl-mediated regulation of Ctr1A varies between tissues. Sima (the *Drosophila* homologue of Hif-1α) is controlled by Vhl in the thorax, while cnc-mediated Vhl activity in the abdomen. We propose that Vhl negatively regulates both sima and cnc, that these transcription factors interact to regulate Ctr1A, copper uptake, and consequently melanin formation, and that differing transcriptional and posttranslational regulatory pathways are active in the fly thorax and abdomen, respectively.^193^

Given the strong conservation of both Slmb and Vhl with their mammalian orthologues, the fruit fly shapes as an excellent *in vivo* system to further probe the relationship between the UPS and copper homeostasis machinery. It will be particularly interesting to determine whether a feedback loop exists whereby copper levels regulate Slmb/Vhl activity. Identification of the E2 ubiquitin conjugase(s) working together with these E3 ubiquitin ligases is a high priority, as is the characterization of the E3(s) directly regulating post-translational down regulation of Ctr1A in response to sima and cnc activation.

Proteasomal regulation of CTR has also been observed in mammalian cells. For instance, loss of CTR1 promoted the proteasomal degradation of CTR2 in HEK293T and OVCAR8 cells, indicating that CTR1 is essential to stabilize CTR2^199^ and that CTR1 and CTR2 function interdependently to regulate copper homeostasis. However, knockdown of CTR2 did not alter the level of CTR1.^199^ Application of the anti-tumor drug cisplatin (CDDP) triggers the rapid polyubiquitination and proteasomal degradation of CTR1 in a mechanism dependent on the chaperone Atox1.^200^ When the Atox1^+/+^ and Atox1^−/−^ fibroblasts were treated with CDDP, only the wild-type fibroblasts showed a CDDP-induced downregulation of CTR1. The E3 ubiquitin ligases required for downregulation of CTR1 and 2 are yet to be identified.

In humans, copper transporters ATP7 and ATP7B play a critical role in copper transport in TGN network and result in Menkes disease or WD, respectively, when mutated. The copper metabolism MURR1 domain protein 1 (COMMD1), a scaffold protein, regulates the folding, stability, ubiquitination, and proteolysis of its interaction partners, which include ATP7A and ATP7B.^201,202^  *Commd1* was originally identified as the gene underlying non-Wilsonian copper toxicosis in Bedlington terriers. This disease is characterized by hepatic copper accumulation resulting in liver fibrosis and eventually cirrhosis.^203–205^ It has been reported that COMMD1 regulates hepatic copper export by interacting with ATP7B^206–208^ since ATP7B is responsible for exporting excess copper in the liver. Consistent with this, copper dramatically accumulated in the liver in liver-specific *Commd1* knockout mice fed with copper-enriched diet.^201^ ATP7A normally resides in the TGN and translocates to the PM only when intracellular copper levels increase. Mutations in the C-terminal DKTG motif of ATP7A block this copper-induced translocation.^209^ Interestingly, COMMD1 restores the expression and subcellular localization of misfolded ATP7A mutants while promoting the proteolysis of misfolded ATP7B proteins,^209^ demonstrating that COMMD1 participates in regulating intracellular copper homeostasis via ATP7A and ATP7B.

**Fig. 5.**
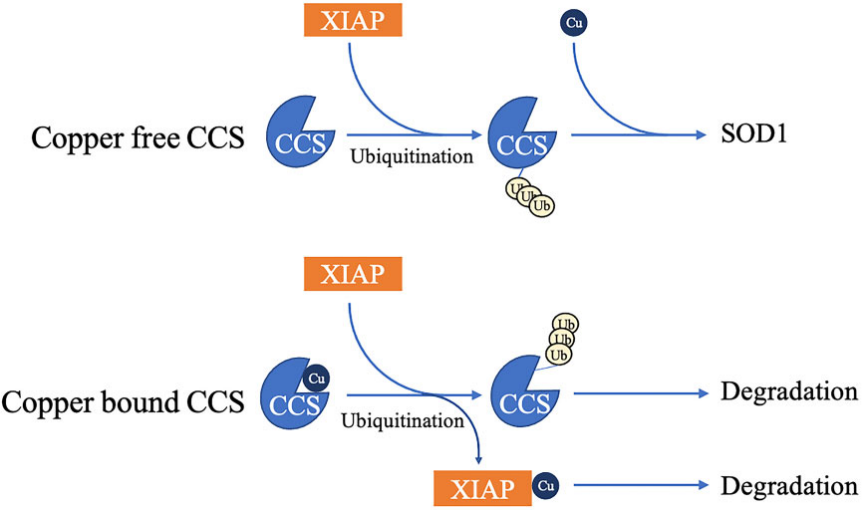
Regulation of copper chaperone CCS by XIAP mediated ubiquitination. Copper-free CCS is ubiquitinated by XIAP to form the nondegradative ubiquitinated CCS, and then the ubiquitinated CCS delivers copper to SOD1. Copper-bound CCS initially transfers copper to XIAP, and then CCS is ubiquitinated. Finally, ubiquitinated CCS and copper-bound XIAP are both targeted for proteasomal degradation.

X-linked inhibitor of apoptosis (XIAP) possesses E3 ubiquitin ligase activity and regulates the ubiquitination and proteasomal degradation of COMMD1.^210,211^ An increase in cellular XIAP levels by ectopic expression results in a decrease in COMMD1 levels, while COMMD1 levels increase when XIAP levels are suppressed,^212^ suggesting that XIAP regulates copper homeostasis in addition to its well-known role as a potent suppressor of apoptosis.^199^ XIAP was found to be a strong copper binding protein and copper-binding led to a conformational change of XIAP, reducing its inhibitory effect on caspase3 activity.^212^ The copper chaperone CCS is responsible for delivering copper to XIAP in mammals and can itself be ubiquitinated by XIAP.^213^ Interestingly, this ubiquitination enhances CCS's ability to deliver copper to SOD1 rather than triggering its degradation. As shown in Fig. 5, Brady *et al*. proposed that interaction of copper-free CCS and XIAP results in nondegradative ubiquitination of CCS and then the ubiquitinated CCS delivers copper to SOD1. In contrast, copper-bound CCS initially transfers copper to XIAP and then CCS is ubiquitinated and targeted for proteasomal degradation.^213^ However, the mechanism underlying why XIAP-mediated ubiquitination enhances CCS ability to deliver copper to SOD1 remains to be investigated.

COMMD1 has also been found to suppress SOD1 homodimerization, which results in a decline in SOD1 scavenging activity and consequently an induction of toxic superoxide anions.^202^ Additionally, many components of the UPS have been identified to regulate SOD1 via post-translational regulation: (i) Mahogunin ring finger-1 (MGRN1, E3 ubiquitin ligase) facilitated clearance of toxic mutant SOD1 inclusions;^214^ (ii) Dorfin (a RING finger-type E3 ubiquitin ligase) physically bound and ubiquitinated SOD1 mutants but did not affect the stability of the wild-type SOD1; (iii) Cdc48 (20 s proteasome complex) degraded SOD1 in a ubiquitin independent manner;^215^ and (iv) Ataxin-3 [a specific deubiquitinating enzyme (DUB)] promoted mutant SOD1 aggresome formation by modifying K63-linked polyubiquitin chains.^216^ Given that one of the important SOD1 functions is buffering copper levels, these data indirectly suggest that UPS components are involved in regulating copper homeostasis.

DUBs reverse the action of E3 ubiquitin ligase complexes by removing ubiquitin or ubiquitin-like molecules from target proteins.^217^ Ubiquitin carboxy-terminal hydrolase L1 (UCHL1) is a DUB that binds and stabilizes monomeric ubiquitin.^218^ A rare inherited form of PD, PARK5, is caused by a missense mutation in UCHL1.^219^ Inhibition of the UCHL1/PARK5 pathway was shown to rescue the copper dyshomeostasis caused by defective ATP7A, placing it downstream or parallel to ATP7A.^220^ In *Drosophila*, ATP7 overexpression mimics the systemic effects of Menkes disease by decreasing cellular copper, while knockdown of ATP7 mimics the systemic WD copper accumulation due to decreased copper efflux.^221^ Changes to ATP7 expression in dopaminergic and serotoninergic neurons make animals more susceptible to death induced by copper feeding. Knockdown of *Uchl* showed protective effects in dopaminergic neurons sensitized by ATP7 up or downregulation,^220^ suggesting that UCHL1 might participate in ATP7 posttranslational regulation.

#### Copper and UPS activity

The UPS participates in protein quality control to prevent the accumulation of non-functional and misfolded proteins.^222^ Interestingly, the activity of the UPS may also be affected by copper homeostasis since some components of the UPS are copper-dependent enzymes, such as E2 ubiquitin enzyme UBE2D2. UBE2D2 is a cuproprotein with two putative copper binding domains, and our results demonstrated that mutations in the copper binding region could cause partial loss of UBE2D2 activity.^223^ In addition, Cu^2+^ ions can target the aggregation-prone regions of ubiquitin and induce its self-oligomerization.^224^ GSH plays a central role in maintaining intracellular copper levels by sequestering and storing excess copper.^4^ In dopaminergic PC12 cells, decrease in cellular GSH leads to reduction of E1 activity which subsequently disrupts the ubiquitin pathway.^225^ There is also some evidence that proteasome activity is subject to copper levels. Xiao *et al*. demonstrated that copper ions inhibit 20S proteasome activity and induce cell death and that the 20S proteasome is able to reduce Cu^2+^ to Cu^+^.^226^ Copper complexes were also demonstrated to inhibit the activity of the 26S proteasome *in vitro* and *in vivo*.^227,228^ Therefore, we propose that the copper homeostasis machinery and the UPS work interdependently to maintain numerous cellular processes.

## Conclusions

Accumulating evidence has revealed that copper homeostasis and the UPS are dysregulated in neurodegenerative disease such as AD, ALS, and PD. Whether this dysregulation is the cause or consequence of the neurodegenerative diseases remains to be proven in most cases. Some studies reported that the accumulation of Aβ42 (a hallmark of AD) or α-Syn (a hallmark of PD) is linked to the disruption of copper homeostasis and UPS activity. This review highlights evidence that implicates the involvement of both the UPS and copper in the development of AD, PD, and ALS. Recently, UPS-associated drugs (e.g. PROTACs) and copper complexes [e.g. Cu(atsm)] have been applied to the therapeutic treatment of AD with promising results, although limitations still need to be overcome. Copper homeostasis involves many molecules such as the transporters ATP7A/B and CTR1 and chaperones Cox17, Atox1 and CCS. Some of these proteins are post-translationally regulated by the UPS, suggesting that it might play a role in maintaining copper homeostasis. Moreover, dysregulation of cellular copper levels could also affect the activity of the UPS, indicating that copper homeostasis and the UPS are interdependent. Therefore, we postulate that these two networks might contribute synergistically to the occurrence of neurodegenerative diseases. If our hypothesis is correct, fully elucidating the relationship between copper homeostasis and the UPS during neurodevelopment will be a critically important step in the search for novel therapeutic candidates for the treatment of neurodegenerative diseases.

## Data Availability

There are no new data associated with this article.
